# Rational assembly of synthetic marine biofilm community with chitinase production

**DOI:** 10.21203/rs.3.rs-9419003/v1

**Published:** 2026-06-04

**Authors:** Diptee Chaulagain, Bristi Paul, Skylar Leslie, Maria Artigues-Lleixà, Maria Pol Cros, Lorena Toloza, Zeyu Tang, Adam Hoover, Marc Güell, David Karig

**Affiliations:** Clemson University; Clemson University; Clemson University; Pompeu Fabra University; Pompeu Fabra University; Pompeu Fabra University; Clemson University; Clemson University; Pompeu Fabra University; Clemson University

## Abstract

Highly diverse multispecies biofilms are ubiquitous in microbial ecosystems; however, our current understanding of biofilm dynamics is limited to single species or low richness studies. We aimed to design a multispecies biofilm with a targeted function, chitinase production, using natural marine bacteria. We present a top-down assembly approach to design functional biofilm communities. Using our method, we found that final community membership was established within 24 hours, regardless of nutrient availability. However, cultivation in nutrient-rich media enabled rapid identification of the competitive dominant taxon, *Pseudoalteromonas*, among the 17 initial isolates used in the assembly. By repeating community assembly in a low-nutrient medium without these highly competitive taxa, we achieved the highest species diversity in the biofilm. The resulting multispecies biofilm exhibited chitinase production and maintained ~ 50% persistence during peak invasion. By comparison, a single species chitinase-producing biofilm formed lower biomass and suffered higher displacement during invasion. Importantly, one member that withstood invasion challenge in the multispecies community was completely undetectable at seven days post-invasion as a single species biofilm, indicating collective invasion resilience in the multispecies community. Further evidence of cooperation for coexistence is supported by increased β-N-acetylglucosaminidase, enzyme that hydrolyzes chitin oligomers, in the 14-member community at later timepoints, while the detected exochitinase activity remained stable. Our findings present a streamlined strategy to assemble diverse and functional biofilm communities for targeted biofilm engineering in marine and applied microbiome contexts, and our achievement of engineered function using natural bacteria offers a powerful complement to synthetic biology.

## Introduction

Most natural and artificial surfaces in the environment are rapidly colonized by bacteria, which, over time, multiply to form biofilms. Bacteria secrete extracellular polymeric substance (EPS) that forms a component of biofilm^1–3^. The structure and interaction in biofilm communities benefit bacteria by enhancing access to nutrients in an often nutrient-limited environment, maintaining extracellular enzymatic activities and protection from antibiotics, toxins, and predation^4–7^. Multispecies bacterial biofilms are common in nature. Culture-dependent taxa identification generally underestimates marine biofilm diversity, with < 100 species identified^8,9^. In contrast, high-throughput sequencing has revealed a median operational taxonomic unit (OTU) richness of 2,790 and a total accumulation of > 25,000 OTUs in a comprehensive study of 101 marine biofilm samples^10^. In the same line, biofilms on artificial surfaces, for example, hospital surfaces and medical devices, are also highly complex, often contributing to hospital-acquired infections and antimicrobial resistance^11,12^. Conversely, the synthetic biofilm communities used to study biofilm characterization and dynamics are limited to a few species, mostly single species of clinical relevance^13–15^ or ranging from two to six taxa^16–20^. Low-diversity communities allow easy manipulation in the lab to understand general underlying principles; however, higher diversity ensures resilience in environmental applications.

Biofilms formed by single species are limited to the characteristics of that organism, whereas a multispecies community can work together in unique ways to provide nutritional, structural, and adaptational benefits not present in a single organism^20,21^. In addition, biofilm properties cannot be predicted solely from the dynamics of planktonic communities because biofilms have different properties compared to free-living or planktonic cells, thereby necessitating biofilm-specific studies. Continued growth and detachment are unique to equilibrium in biofilm communities and vary with species in the community. A four-species bacterial biofilm using soil bacteria developed under flow reached steady biomass after 30 hour of growth^18^. Interspecies interactions guide the spatial assembly and function of the biofilm, not only by influencing member composition, but also by modulating the biofilm matrix^20^. For example, a core set of functional genes was identified in the bacterial community associated with green microalga *Ulva australis*, despite high phylogenetic variability, suggesting functional traits rather than species composition determine the community assembly^22^. In a multispecies community, redundancy of key functions can help community resilience, where a key role may be satisfied by different members under different environmental conditions^23^. Furthermore, a diverse community has access to a broader spectrum of resources than a monospecies, providing increased resistance to invasion^16^.

Synthetic biofilm communities on inanimate objects can have a wide range of applications, from mitigating biofouling by drag-reducing organisms on marine vessels, treating wastewater, and reducing medical risk from pathogenic biofilms in medical devices. The retention of enzymes in the biofilm matrix can extend the activity and turnover of enzymatic processes^24,25^. The prolonged release of matrix-embedded enzymes in biofilms can be beneficial for sustained environmental applications compared to free-living cells or purified enzymes. However, the design of such functional synthetic biofilm communities requires standard methods for biofilm community assembly and the understanding of the dynamics and stability in biofilm communities. The gap between the number of taxa *in vitro* studies and *in vivo* biofilms, detailed above, is likely due to a lack of standard methods to develop and study diverse biofilm communities. The design of functional biofilms suitable for deployment remains challenging due to the overwhelming number of potential interactions among multispecies community members, environmental taxa, and the compounds and conditions of the environment.

We address the methodological gap by combining a top-down community assembly strategy with high throughput experimental methodology to test a variety of biofilm communities on desired materials. The top-down assembly of bacterial communities involves mixing bacteria with targeted traits, followed by serial passage or selection in the presence of the desired stressor to obtain a stable biofilm community. Using top-down assembly, we constructed a synthetic biofilm community using natural bacteria from the marine microbiome. The candidate community members were selected based on the desired traits for which the measurement techniques are available to us, namely, adhesion, chitinase secretion, and colonization. Our approach rapidly identifies specific taxa that strongly impacts community diversity and allows inclusion or exclusion of these taxa to obtain desired diversity levels. Applying the top-down biofilm assembly method in nutrient-rich medium, we identified *Pseudoalteromonas* as the dominant taxon referred to here as “bullies,” which reduce diversity in multispecies biofilms through a combination of metabolic competence and direct inhibition. We then demonstrate that excluding the bullies from the initial assembly yields a diverse, multispecies biofilm community with chitinase activity that is able to persist during invasion. Our work enables biofilm engineering by enhancing capabilities for multispecies biofilm studies and providing insight into the dynamics of biofilm community assembly, which can be extended to other functions or application environments by appropriately sourcing microbes from the target environment.

## Results

### Selection of traits and taxa

We aimed to establish a method for top-down biofilm community assembly and study the dynamics of a multispecies biofilm community on an inert substrate, polyvinyl chloride (PVC) (Fig. 1). PVC is the third most widely used plastic globally and is used in construction, plumbing and electrical insulation^26^. Towards the first objective of establishing a standard method for top-down biofilm community assembly, we determined (i) adhesion, (ii) chitinase production, (iii) colonization resistance, and (iv) drag reduction as general traits of interest for the synthetic marine biofilm community based on a literature review. Adhesion is the first step of biofilm formation, thus an important trait for a biofilm community. The chitinase enzyme, commonly known for its enzymatic degradation of chitin, has other ecological functions beyond its role in the metabolic process. Chitinase can degrade chitin in barnacle adhesive and provide colonization design process involves trait identification, member selection and trait integration by establishing a multispecies community through top-down method of community assembly. resistance against barnacle settlement in marine environments^27^, whereas invasion resistance by other bacteria can arise from quorum sensing regulated chitin metabolism^28^, antibiotic production^29^, and competitive resource exploitation^16^. Natural biofilms are subjected to invasion as well as predation, for example by the predatory *Bdellovibrio*, which mainly targets gram-negative bacteria^30^. A mixed community of gram-positive and gram-negative bacteria is likely to better tolerate and recover from predation. Additionally, biofilm and marine biofouling, increases drag in marine vessels^31^. Thus, drag reduction was selected as an advantageous trait for a beneficial synthetic biofilm. The marine bacteria containing the traits of interest were identified and obtained through the search for homologous proteins (see methods). A complete protein system for any of the four selected traits was identified in 3,957 genome assemblies encompassing 738 genera, 44.07% of which contain more than one desired trait (Fig. 2a and 2b; Supplementary Table 1).

To select candidate community members, we focused on three measurable traits within our laboratory facility - adhesion, chitinase production, and colonization resistance. The members were selected using genomic trait search and/or experimental verification of these functions as a single isolate, permitting redundancy (Table 1). Efficient biofilm-formers were identified in an adhesion assay using crystal violet staining in a previous study^32^. The chitinolytic isolates were obtained from our previous screening^33^. The Roseobacter clade is abundant in marine water and is the dominant taxa in primary surface colonization^34–36^. Additionally, the Roseobacter clade isolates we selected have established genetic manipulation procedures^37^ that allow the augmentation of function if desired in future work. The 17 isolates selected for the synthetic community include 10 genera and 13 species (Table 1). The subsequent use of 17-member community defines a community initiated with all members in Table 1. We kept more than one isolate from the same species only if the isolates demonstrated a difference in the selected feature, so that redundancy captured meaningful functional variations. For example, *Pseudoalteromonas piscicida* ATL3 and *P. piscicida* ATL28 have differences in chitinolytic activity^33^. *Pseudoalteromonas* sp. ATL1, along with the two *P. piscicida* isolates ATL3 and ATL28, is referred to hereafter as the “Pseudoalteromonas ATL group”. *Psychrobacter piscatorii* isolates (H25, H26, H27 and H29) formed different biofilm biomasses, and a three-member community of H13, H22 and H27 produced significantly higher biofilm biomass compared to single species and other three-member communities tested^32^. Additionally, any isolates with identified pathogenic genes, for example, *Vibrio fluvialis*^33^, despite the presence of the desired biofilm traits, were excluded from the community design.

### Development of culture-based identification of members of the synthetic biofilm community

A broad challenge to microbial community analysis and engineering is the lack of a method for quantifying community composition in a manner that combines cost and speed practicality with the ability to determine absolute levels of viable members with high taxonomic resolution. We developed an approach for quantifying absolute population levels of viable members based on a combination of selective inhibition and colony morphological features. For efficient screening of inhibitor compounds, we used Biolog (Biolog Inc, USA) phenotyping microarray plates to screen for 288 unique chemicals for each isolate (see methods; Supplementary Fig. 1a, 1b and 1c, Supplementary method).

We identified unique selective conditions combining chemical selection and morphological features for 11 out of 17 isolates (Fig. 2c and 2d; Supplementary Table 2). ATL3 and ATL28 are identified as one unit. Similarly, B2 and B11 are identified as one unit. Altogether, this method can identify the presence of 15 out of 17 isolates except H13 and H27. The sequence analysis of V3-V4 region of 16S rRNA gene and full 16S rRNA gene sequence shows that two *P. piscicida* isolates (ATL3 and ATL28) are identified as one, similarly, the *P. piscatorii* isolates (H25, H26, H27 and H29) are identified as one unit. Thus, for our selected community members, where full 16S sequence analysis would not lead to differentiation of all isolates, we used chemical selection, which quantifies only the viable cells from biofilm, yields absolute population data, and takes less time than DNA sequencing-based approaches.

### Establishment of biofilm growth and analysis

We used coupons in multi-well plates for biofilm growth, which facilitates the testing of multiple samples (Fig. 2e). Coupon sizes can be chosen to provide either horizontal or slanted surfaces for colonization. We tested both configurations by monitoring the biofilm growth of a 17-member community in Marine Broth (MB). In the horizontal position, biofilm coverage grew quickly within 2–3 days, followed by detachment and regrowth within a 7-day test period (Fig. 2f). By contrast, for slanted coupons, a progressive increase in biofilm coverage was observed over a week (Fig. 2f; Supplementary Fig. 1d). Based on these results and the fact that the slanted configuration makes it easier to distinguish true biofilm content from settled planktonic growth, we chose to use the slanted configuration for subsequent experiments. Specifically, we slanted 10×13×0.5 mm coupons in 24-well plates and submerged them using 1.2 mL of media.

### Top-down assembly identifies the media effect and dominant species

We performed top-down assembly with the selected 17 isolates (Table 1) using three media types, MB, Väätänen Nine-Salt Solution (VNSS)^42^ and 1% MB in artificial seawater (ASW)^43^, using nutrient availability as a stressor. In nutrient-rich media, MB and VNSS, only two out of 17 isolates were detected in the biofilm at any time point; studied at 1, 3, 5 and 7 days (Fig. 3a and b). *P. piscicida* (ATL3 or ATL28) was identified as the dominant species, and *Phaeobacter sp*. (B27) was identified at a lower abundance by ~ 1000X (Fig. 3a and b). However, in low-nutrient media, 1% MB in ASW, six additional isolates, including four isolates selected for adhesion, one isolate selected for chitinase activity and additional *Pseudoalteromonas*, ATL1, were detected (Fig. 3c). Interestingly, the total abundance of Pseudoalteromonas ATL group and non-Pseudoalteromonas species were similar in the biofilm community assembled in 1% MB in ASW (Fig. 3d). To prevent erroneous estimation of community variation by an individual metric, we performed principal coordinate analysis (PCoA) using three distance measures. The selected methods (Bray-Curtis, Morisita-Horn and Jaccard) balance abundance, sensitivity to rare taxa and presence/absence. For the comparison of treatments, such as media or time, a Permutation-based Multivariate Analysis of Variance (PERMANOVA) was applied to the distance metrics, followed by Bonferroni correction for multiple comparisons. A consensus effect of media (nutrient richness) in the community beta diversity was obtained with all three metrices (PERMANOVA; [Pr(> F) = 0.001]), whereas the pairwise analysis revealed no significant difference between nutrient-rich MB and VNSS (Fig. 3e, Supplementary Fig. 2). In low-nutrient media, the *P. piscicida* dominance was lost and replaced by a similar abundance but higher diversity of non-Pseudoalteromonas isolates (Fig. 3a, b, c and e).

### Multiple factors contribute to the “bully” effect

We define the competitive dominance of a species in a multispecies community, conferred by multiple factors including metabolic capability, selective inhibition, and phylogenetic relatedness, as the “bully” effect. First, we tested whether dominance in the synthetic community could be exclusively due to metabolic competition, leading to the monopolization of nutrients by fast-media pair was also analyzed using PERMANOVA, applying Bonferroni correction for pairwise analysis. Refer to Supplementary Fig. 2a and 2b for analysis based on Morisita-Horn and Jaccard dissimilarity, respectively.

growing species while passively suppressing slow growers, also known as the Jameson effect. We performed a growth assay of the 17 isolates individually in all three media types. Low nutrient conditions in 1% MB in ASW resulted in lower biomass as observed by low overall OD600 in the kinetic growth assay, as expected (Supplementary Fig. 3a). Most of the isolates also have a higher maximum growth rate in nutrient-rich MB and VNSS compared to 1% MB in ASW (Fig. 4a). Interestingly, some isolates have a similar maximal growth rate in all three media, indicating that they have optimum resource utilization even in low nutrient conditions but cannot achieve high biomass due to quickly exhausting the limited resources. We did not detect a significant difference in the lag phase length by media type, except for two isolates, ATLC8b which had a longer lag phase in VNSS, and B13, which had a longer lag phase in 1% MB in ASW (Fig. 4b).

#### member of the community.

**(a)** maximum growth rate (μmax) and **(b)** length of lag phase (λ) of the 17 isolates in three different media; 1% MB in ASW, VNSS and MB. The error bars indicate standard deviation for n = 3, with two technical replicate for each biological replicate. See Supplementary Fig. 2 for individual growth curves **(c)** Phylogenetic relatedness of the 17 isolates used in the community with the heat map of interaction of *Pseudoalteromonas* and other community members in agar co-culture. The phylogenetic tree is the compressed tree drawn with Mega 11 using maximum likelihood method and 1000 bootstrap (see Supplementary Fig. 3B for scaled branch length and full description). Fold change (FC) in colony area of row species are shown in presence of column species with respect to growth alone. FC = 0 (complete inhibition or no growth), FC > 0 < 1 (partial inhibition), FC = 1 (no effect), FC > 1 (promotion). A fold change of < 0.5 (> 50% inhibition of growth) is marked by asterisk. Enhancement was observed only on 1% MB agar medium for two species; B13 in presence of ATL1 and ATL3 and ATL28 in presence of ATL3 with maximum fold change of 1.4.

A close phylogenetic relatedness often means niche overlap and stronger competition for resources. In a pairwise inhibition study using soil bacteria, Qiao et al. found the strongest inhibition between phylogenetically close members at both high and low nutrient conditions^44^. Co-culture using spot assay on agar plates revealed that *Pseudoalteromonas* sp. ATL1 inhibited most of the isolates except *Phaeobacter gallenciensis* (B13) in nutrient-rich half marine agar (hMA), with more than 50% inhibition for 11 isolates (Fig. 4c, Supplementary Fig. 3b). *P. piscicida* (ATL3) inhibited similarly to ATL1; however, the extent of inhibition was weaker (< 50%) in *Psychrobacter* species (H25, H26, H27 and H29). Interestingly, in low-nutrient conditions prepared by adding agar to 1% MB in ASW to measure colony growth, the growth enhancement of *P. gallenciensis* (B13) by *Pseudoalteromonas* sp. ATL1 and by *P. psicicida* ATL3 was observed. In 1% MB in ASW agar, a less severe inhibition for most taxa relative to hMA was observed without fully relieving the inhibition effect despite a significantly longer lag phase of the Pseudoalteromonas ATL group, indicating an additional inhibitory mechanism besides nutrient competition. The members of Pseudoalteromonas ATL group are also capable of inhibiting phylogenetically close *Simiduia agarivorans* ATLC8b (> 50% inhibition by both ATL1 and ATL3 in hMA and only by ATL1 in 1% MB in ASW agar) and *Rheinheimera* sp. ATLC3 (Fig. 4c, Supplementary Fig. 3b and 4). Thus, Pseudoalteromonas ATL group is identified as bully, achieving dominance through fast growth and selective inhibition.

### Excluding dominant members, bullies, from the community enhances diversity

By assembling communities in rich media, we identified the dominating species, *P. piscicida*. As all the Pseudoalteromonas ATL group isolates had high inhibition towards other taxa (Fig. 4c), we excluded all three isolates in the *Pseudoalteromonas* genus to build a community without bullies (Supplementary Fig. 5a). This community, initiated with 14 isolates, referred to as the 14-member community hereafter, in low-nutrient 1% MB in ASW, resulted in eight stable community members. Stable members are defined as the members detected at all time points. Among the eight stable members, three were chosen for adhesion/biofilm formation, three were chosen for chitinase production, and two were chosen for primary colonization/antibiotic production (Fig. 5a). *P. piscatorii* H29 was detected close to the detection limit and was detected only on day 1 in all three replicates (Supplementary Fig. 5b). The non-Pseudoalteromonas species diversity is higher (eight stable isolates) in the 14-member community compared to three stable non-Pseudoalteromonas isolates in the 17-member community (Fig. 3c).

Morisita-Horn and Jaccard dissimilarity, respectively. **(c)** Total abundance has an increasing trend but not significantly different among four time points between one and seven days indicating slow biofilm growth (one way ANOVA with Tukey pairwise test). **(d)** A separate experiment carried out for 14 days shows significant increase in total abundance at 14 days compared to the first two timepoints (One way ANOVA with Tukey pairwise comparison; * indicate p < 0.05).

### Temporal effect on biofilm diversity and growth

After confirming the effects of media and taxa on biofilm diversity, we examined the temporal effect on the biofilm communities. The principal coordinate analysis using Bray-Curtis, followed by PERMANOVA analysis using the days post inoculation as effect, displayed no significant (Pr(> F) = 0.664) clustering in the 14-member community (days 1, 3, 5 and 7) (Fig. 5b). Consensus result for absence of day effect was obtained by applying Morisita-Horn and Jaccard dissimilarity metric (Supplementary Fig. 5c and 5d). Furthermore, the absence of a replicate effect in the diversity of the 14-member community (BrayCurtis dissimilarity and PERMANOVA analysis; Pr(> F) = 0.664) (Supplementary Fig. 5e) supports the absence of a temporal effect, excluding any role of replicate variation in the observed results. Similar analysis of the 17-member community for the temporal effect confirmed the absence of a day effect in the 17-member community tested in three different media (Bray-Curtis dissimilarity and PERMANOVA analysis; Pr(> F) = 0.892) (Supplementary Fig. 5f).

In addition, the total abundance of all isolates in the 14-member community had an increasing trend but not a statistically significant increase in one week old biofilm (Fig. 5c). Thus, we performed the experiment for two weeks, confirming a significant increase in total abundance at Day 14 (Fig. 5d) compared to Day 1 and Day 3 (One-way ANOVA; Tukey multiple pair comparison; p < 0.05) while the isolates identified remained the same as Day 7. The optical coherence tomography (OCT) displayed a consistent outcome with total abundance, as a higher number of patches and biofilm coverage were observed in later timepoints of Day 7 – Day 14 (supplementary Fig. 5a). Furthermore, the total abundance in the biofilm initiated with the 14-member community was higher compared to a single isolate biofilm (Supplementary Fig. 5g) confirmed in three single isolate biofilms, H2, ATLC3 and B13, one from each desired functional category, adhesion, chitinase production and antibiotic-producing early colonizer, respectively.

Thus, our data collectively indicates that biofilm community members are selected within 24 hours in all media types, and overall cooperative behavior in a multispecies community promotes biofilm biomass in low-nutrient media.

### Synthetic community without bullies can produce chitinase and persist during invasion

In addition to confirming the presence of chitinase-producing members in the 14-member synthetic biofilm community by absolute quantification, we verified the integration of chitinase activity using a chitinase assay. Based on whole genome sequence analysis, *Rheinheimera* sp. ATLC3 had only one chitinase gene (endochitinase), whereas *S. agarivorans* ATLC8b and *I. dokdonensis* ATLC11 each had two genes encoding glycosyl hydrolase family 18 chitinase^33^, which can have both endo- and exo-chitinase activity^45^. We used the three substrates in the chitinase fluorometric assay kit (Millipore Sigma; CS1030) to test endochitinase, exochitinase (chitobiosidase) and β-N-acetylglucosaminidase (NAGase) activity. Endochitinase hydrolyzes the chitin chain randomly at internal sites, exochitinase releases monomers and dimers from the ends of chitin polymer, and NAGase produces monomers from dimers and short oligomers. Only chitobiosidase and NAGase activities were detected, with low levels of chitobiosidase activity at all days tested and the highest NAGase activity in 14 days old biofilm (Fig. 6a). Interestingly, the total cell abundance is also highest at day 14, indicating higher biofilm cell density contributing to high NAGase activity; however, the total abundance of detected chitinase producers (ATLC3, ATLC8b and ATLC11) displays increasing trend but not significant increase from day 1 to day 14 (Supplementary Fig. 5h). It is important to consider that the substrate (4-Methylumbelliferyl N-acetyl-β-D-glucosaminidine) can detect all NAGase activity, including that of chitinase and NAGsae. In a single isolate biofilm, ATLC3 exhibots low level of endochitinase activity, as expected; however, NAGase activity was absent (Fig. 6b), indicating that non-chitinase producers can benefit from the chitinase activity of chitinase producers, resulting in the chitin oligomers and dimers. NAGase activity was verified in a 14-day-old single-member biofilm of two isolates, H02 and B13, which lacked chitinase activity on chitin agar plates (Fig. 6c). In a model soil consortium consisting of chitin-degrading and non-degrading members, the most abundant members under chitin degradation environment were the ones that could take advantage of the chitin degradation products rather than the chitin-degraders^46^.

biofilm of the B13, ATLC3 and H02 with the mix of the three *Pseudoalteromonas* species shows persistence of native species only in B13 biofilm. Biofilm growth medium: 1% MB in ASW. Unit definition: One unit of chitinase activity release 1 mmole of 4-methylumbelliferone from the appropriate substrate per minute at pH 5.0 at 37°C.

The invasion of the 14-member three days old biofilm community by a mix of bullies (three *Pseudoalteromonas* isolates), resulted in the loss of only two stable members, ATLC8b and ATLC11, which are both chitinase-producing isolates. The highest abundance of the Pseudoalteromonas ATL group was observed at five days post-invasion (53% total abundance). Surprisingly, the non-Pseudoalteromonas abundance increased significantly, reducing the Pseudoalteromonas ATL group abundance to less than 20% on seven days post-invasion (Fig. 6d). Interestingly, the increase in non-Pseudoalteromonas abundance was caused by a sharp spike in *P. gallensiensis* B13 density on seven days post-invasion. At the monospecies level, the invasion of single-member ATLC3 biofilm by the bullies resulted in the displacement of native species to an undetectable level, and the invasion of H02 biofilm resulted in > 95% loss of native species (Fig. 6f). However, the B13 biofilm recovered from initial dominance of invaders observed up to three days post-invasion, limiting invaders to less than 50% at five- and seven-days post-invasion (Fig. 6f).

### Biofilm community assembly is guided by multiple factors

Material surface properties can affect the initial adhesion, primary colonization and biofilm growth^47,48^. Studies of monospecies biofilms show that hydrophobic bacteria have higher adhesion and biofilm formation on hydrophobic surfaces and vice versa^47,49^. The PVC material used in this study is weakly hydrophobic (water contact angle [°] = 90.77 ± 5.67) (Supplementary table 3). Among the 17 isolates, only H02 is hydrophobic, with a measured water contact angle > 45° for the bacterial colony^50^. However, H02 is not a dominant species at the earliest time point of 24 hours in both the 17-member and 14-member communities and up to the 14-day study period (Fig. 3c and 5a). In addition, H02 does not display significantly higher abundance at 24 hours or higher biofilm biomass on tested days from days 3 to 14 compared to two other single isolates in which the experiment was carried out, B13 and ATLC3 (Supplementary Fig. 5g). Thus, our study on marine biofilm indicates that media and species interaction have a greater influence on bacterial adhesion and biofilm formation than the material's physical property of hydrophobicity.

## Discussion

Studies of multispecies microbial community dynamics so far have largely focused on planktonic growth^51,52^. However, biofilms develop new properties that cannot be predicted from planktonic cell behavior^53^. We established a biofilm growth and member identification method investigating the biofilm properties of a synthetic community initiated with 17 members selected systematically to integrate desired traits. We demonstrated that the biofilm community members are selected within 24 hours, guided by nutrient availability (media) and initial species composition, and there is an absence of temporal effect in diversity up to a week (Fig. 3, 4, and 5), suggesting stability under the tested conditions. This absence of temporal effect was observed in both nutrient-rich and limited media, indicating that media affects initial adhesion and member selection within 24 hours. However, once members are selected, there is no temporal effect specific to a particular media type.

We did not find a significant effect of surface properties, namely wettability/hydrophobicity, in determining early colonizers (species adhering at the first 24 hours of exposure) or shaping the final community composition. The most hydrophobic member, H02, was undetected in rich media and was not a dominant taxon in nutrient-limited media. Sushmitha et al (2021) similarly found that the early stage of succession in natural marine biofilm was shaped by temporal succession rather than the substrate on which the biofilm is growing^54^. The temporal succession observed in the natural environment is most likely due to the constant influx of new taxa in the field. Our initial implementation has a bias towards using higher nutrients and sterile media replacement compared to natural seawater. However, as we demonstrate, the synthetic biofilm can be strategically exposed to invaders to test invasion resilience.

First, we used nutrient-rich media to identify the competitive dominant taxa, *P. piscicida* ALT3 and ATL28, using top-down assembly and investigated the interaction of *Pseudoalteromonas* with other members. Then, the three isolates of the Pseudoalteromonas ATL group were excluded from the initial 17 members to design a community resulting in higher diversity (Fig. 3c, 4a and 4b). The resulting community exhibited resilience to an invasion challenge with a high density of bullies. Specifically, ~ 50% of the original biofilm composition of a 3-day-old biofilm initiated with the 14 members persisted when exposed to the Pseudoalteromonas ATL group showed the persistence of ~ 50% of the original biofilm composition even at the peak of invasion on five days post-invasion (Fig. 6d). For the upper 200 m of open ocean, the prokaryotic cell density is ~ 5 × 10^5^ cells/mL^55^, yet 1.48 × 10^7^ ± 3.93 × 10^5^ (average ± standard deviation) cells/mL were added for the invasion challenge. Thus, the synthetic bully-free community persisted despite an invasion challenge with a 100X higher load of bullies compared to the average prokaryotic cell density in ocean water. This demonstrates that a rational top-down assembly can be used to design a diverse synthetic community to resist invasion in the environment.

Invasion can lead to temporal differences in natural marine biofilms. In the 14-member biofilm, two members were undetectable on day 1 post invasion, whereas invader abundance increased gradually to ~ 50% of total abundance until day 5 post invasion before plummeting in conjunction with a sharp spike in *P. gallensensis* B13 on Day 7 (Fig. 6e). Interestingly, the Pseudoalteromonas ATL group do not inhibit B13 in the spotting assay, instead show some promotion in B13 colony size (Fig. 4c). Invasion of single species B13 biofilm with Pseudoalteromonas ATL group resulted in invader dominance with ~ 80% invaders but B13 gradually regrew with ~ 40% invaders detected at Day 7. In a coculture of *P. piscicida* and *Phaeobacter sp*. in MB, co-existence is facilitated by the ability of *Phaeobacter sp*. to reduce tropodithietic acid, an antimicrobial compound produced by *P. piscicida*^56^. In our study, we detected the dominance of *P. piscicida* ATL3 and ATL28 over *Phaeobacter sp*. B27 and loss of B13 in the 17-member community in nutrient rich media, whereas growth enhancement of both B27 and B13 in the one-to-one spotting assay (Supplementary Fig. 3b) and recovery of B13 after Pseudoalteromonas ATL group invasion in 14-member (Fig. 6e) and single-member biofilm (Fig. 6f). In contrast, ATLC3 and H02, which stably persist in invasion as members of the 14-member biofilm community (Fig. 6e), become completely undetectable and reduced to 5% of the total abundance, respectively, at seven days post-invasion of single-species biofilm. This suggests that (i) cooperative behavior in a multispecies community can provide invasion protection to some members that lack resilience on their own and (ii) species interaction is affected by nutrients and overall members in the community. Community interactions can also result in emergent properties. For example, a *Streptomyces* sp. was found to develop chitin utilization when present in a chitin-degrading soil consortium^46^. Furthermore, the multispecies community exhibits higher biofilm biomass (Supplementary Fig. 4b), which can assist invasion resilience indirectly through colonization resistance.

Our community is diverse enough to observe both competition and cooperation among the members. Such a relationship between members can be further affected by spatial organization. Similar to our finding, a gut community study showed that a diverse community is highly efficient in protecting against pathogen colonization, compared to single species, which offer negligible benefits^57^. A study of biofilm spatial patterning using two strains of *Escherichia coli* showed competition at spatial proximity in the absence of antibiotics, whereas at an intermediate antibiotic concentration, cooperation was observed, where the resistant strain provided protection to the susceptible strain^7^. A future study combining biofilm spatial patterns with species composition can reveal the detailed ecological relationships and mechanisms of invasion resilience in a diverse community like ours.

We developed a pipeline for a high-throughput, flexible, and repeatable biofilm growth method in multiwell plates using PVC coupons. Furthermore, our absolute identification method, using chemical and morphological methods, provides cost-effective laboratory quantification independent of lengthy sequencing methods that are frequently outsourced. We were able to differentiate between *Psychrobacter* strains with identical 16S sequences using chemical selection (Fig. 2c and Supplementary Fig. 2a). Niu et al. successfully used the chemical selection method for the absolute quantification of a seven-member community^58^. However, the chemical method has limitations for very large communities with more than 20 members, as unique chemical selection can become dependent on the limited chemicals available. We demonstrate that nutrient-rich media can be used to rapidly identify dominant taxa and exclude them to promote higher diversity. If a higher diversity community with the competitive dominant taxa is desired, a low-nutrient medium can be used. In the lowest nutrient condition tested, with 1% MB in ASW, nutrients are quickly exhausted, which likely provides other members with an opportunity to attach and form a biofilm, leading to an increase in biofilm diversity. A combination of low nutrient media and exclusion of competitive dominant taxa, *Pseudoalteromonas*, resulted in the highest diversity in the biofilm while maintaining all desired traits in the community (Figs. 5 and 6). Furthermore, our strategy of using isolates for redundancy ensured that at least one isolate with the desired function was retained in the community, for example, *Psychrobacter* sp. H25 was the only identified stable member among the four initial *Phychorbacter* species used in the 14-member community.

Chitinase quantification confirmed our targeted function of chitinase activity in the designed community. Although members capable of producing endochitinase (ATLC3) and both endochitinase and exochitinase (ATLC8b and ATLC11) were present in the synthetic biofilm, only exochitinase activity was detected in the assay. Interestingly, the NAGase activity increased sharply at later timepoints (Days 10 and 14) despite the lack of significant increase in total abundance of chitinase producers (Fig. 6a, Supplementary Fig. 4e). This observation suggests that chitinase may promote cooperation by supporting members that cannot utilize chitin directly but can use the oligomeric and dimeric chitin breakdown products.

We identified an interesting overlap between chitinase production and adhesion in publicly available marine genomes. Of 1,580 genomes with chitinase systems, 93.6% also harbored traits for adhesion (Fig. 2a and 2b). Chitinase activity has been shown to be important for pathogenesis in virulent species like *Vibrio cholerae*, which may use chitinase for binding and utilizing mucin in the host intestine^59^. Furthermore, chitinase is positively correlated with extracellular DNA secretion and biofilm formation in *Aspergillus fumigatus*^60^, and is a part of the extracellular matrix in *Pseudomonas aeruginosa* biofilm^61^. The exact role of chitinase production in bacterial biofilms is not understood yet. It is intriguing that we detected chitinase activity on biofilm grown on PVC, a non-chitin surface. Thus, our study opens an interesting research avenue for understanding the role of chitinase in multispecies biofilm formation by guiding biofilm composition and architecture.

We demonstrated that our top-down biofilm community assembly approach can yield diverse, stable biofilm communities with targeted functions. This allowed us to study the community dynamics and emergent properties of synthetic biofilm communities with higher richness levels than previous studies. Therefore, our methods offer a powerful tool for understanding even more diverse, natural biofilms under precisely controlled conditions. The top-down nature of our approach permits extension to higher richness levels. Moreover, our work advances biofilm engineering. In the future, the assembly method can be easily adapted to select for resilience by replacing simple passaging steps with chosen stress conditions. Furthermore, integration of our approach with model-guided design optimization will offer a powerful path towards functional biofilms that can tolerate conditions of application environments, rather than simply laboratory contexts.

## Methods

### Trait and species selection

To aid community member selection, a set of traits that facilitate biofilm formation and mainteinance was prioritized: adhesion, chitinase production, colonization resistance and drag reduction. Reference proteins that showcased these traits, containing 27,27, 1 and 2 proteins for adhesion, chitinase production, colonization resistance and drag reduction, respectively, were retrieved to search for homologous proteins in the marine bacteria genomes available (Supplementary Table 4). The Integrated Microbial Genomes & Microbiomes database (IMG/M)^62^ was browsed, and a dataset of bacterial genome assemblies present in marine ecosystems was downloaded on the 30th of January 2023. A total of 4839 genomes were retrieved from the National Center for Biotechnology Information (NCBI) database, accounting for 2,982 different marine bacteria species. The available proteomes were retrieved in addition to the annotated assemblies. The assemblies containing only genomic sequences were annotated with Bakta, a prokaryotic command line annotation tool^63^. To select the species with prioritized traits, HMMER v3.4^64^ was used to search the IMG translated proteomes for proteins homologous to the proteins of the reference systems described with the traits of interest. *hmmscan* was used to extract the domain scaffolds of the reference protein systems, which served as templates to find homologous protein systems in the marine bacteria assemblies. For building the scaffolds, overlapping PFAM^65^ domains within proteins with E-values lower than 10^− 3^ were excluded. *hmmsearch* of the protein system scaffolds was used to find homologs in every proteome, and the results were parsed to obtain a single tabular file with the assembly identifier and the proteins found. As some traits depend on the presence of several proteins at the same time, partial presence of these protein systems in a species is not considered in the results. Of all the 4,479 proteomes present in the dataset, HMMER was able to find complete protein systems in 3,957 (Supplementary Table 5).

### Media and growth conditions

Marine broth (Difco), VNSS^42^ were used. Agar plates contain 1.5% agar in addition to the respective media. Half marine agar was prepared by using half the recommended amount per liter of MB and 1.5% agar. Media with chemical selection were prepared by adding stock chemicals to cooled down media (50–55 C) before pouring plates and the plates were used within a week. We suggest using Difco MB because when we tried MB (Millipore Sigma), it resulted in slow growth in many isolates and the absence of growth in some. Growth and chemical selection should be verified in new media. Artificial seawater (ASW) was prepared according to the Cold Spring Harbor Laboratory protocol^43^. All single isolate growth was carried out at 30 C to harvest enough bacteria for community assembly in overnight cultures. Biofilm growth was carried out at room temperature (21–23 C).

### Development of Selective plating for species identification

All isolates were grown in 3 mL of MB at 30 C/200 rpm until OD600 = 2 ± 0.5; 1 mL of fresh MB was added and continued growing for 4–5 hours. The culture was centrifuged at 3,200g for 8 min to pellet the cells which were then washed by resuspending the pellet in Phosphate Buffer Solution (PBS; pH = 7.4) and centrifuging again to pellet it. The bacterial pellet was resuspended in MB to prepare a targeted OD determined by CFU/mL plating (supplementary Fig. 1) to get a cell density of ~ 10^7 cells/mL using a turbidimeter (Biolog). The Biolog Phenotyping plates (PM11C, PM12B and PM13B tested in triplicates) were inoculated with 100 μl of adjusted culture and incubated at 30°C/200 rpm for four days. Endpoint absorbance at A600 and A750 was measured using Biotek Gen5 plate reader every 24 hours. All three plate types were tested for ATLC3, ATLC8b, H2, H13, H22, H25, H26, H27, H29, B2 and B11. A script was developed to evaluate the inhibition of non-targeted isolates relative to the degree of undesired inhibition of the target to find unique selective chemicals (maximum of chemical combination allowed = 2) for each isolate (Supplementary method). The appropriate dose was determined by using a dose-response curve to find an appropriate concentration of chemicals that inhibit non-target isolates without affecting the growth and recovery of the targeted isolate.

### Top-down community assembly setup for biofilm growth

Bacterial cultures were grown as described above, and then centrifuged at 3,200g for 8 min to pellet the cells. The cells were washed by resuspending the pellet in the targeted community assembly media and centrifuging again to pellet it. MB and VNSS were used as rich media, and 1% MB in ASW as low-nutrient media. The bacterial pellet was used to prepare a cell density of ~ 10^7 cells/mL using a turbidimeter (Biolog) in the targeted community assembly medium. A community mix (inoculum for biofilm) was prepared by mixing an equal volume of cell suspension of each isolate. PVC coupons of 10mm*13mm*0.5 mm were sterilized by dipping in 70% ethanol for 2 minutes, followed by UV treatment of 10 min on each side. The sterilized PVC coupon was placed in a well of 24-well non-treated plates, and 1.2 mL of the bacterial mix was dispensed to immerse the coupon completely. The coupon makes a slant on the well due to its dimensions, allowing biofilm formation on all sides. The biofilm setup was incubated at RT (20–22 C)/120 rpm. The coupons were transferred to new wells containing 1.2 mL of fresh media every 24 hours until biofilm extraction. The sample size was chosen to maintain experimental throughput, as increasing the number of replicates would reduce the testing community in different conditions. At least two replicates were performed where the same community was tested in different media types. The most repeated community, 14-member in 1% MB in ASW, was tested five times.

### Biofilm extraction and CFU/mL plating

For the biofilm collection, the coupon was dipped twice in each of the two wells containing 2 mL of PBS (pH 7.4) to remove loosely attached planktonic bacteria. The liquid droplets attached to the coupon were absorbed by standing the coupon on a sterile paper towel. The two coupons were transferred to a 15 mL conical tube containing 2 mL of PBS (pH 7.4). The biofilm attached to the coupons was extracted by vortex: sonication: vortex method for 2:5:2 minutes using a maximum vortexing speed of 3200 rpm and 43 kHz sonication (Ultrasonics Quantrix 90H; L&R Manufacturer). CFU/mL plating was performed using the previously described plating method^66^ in triplicate in each selective media and hMA. CFU/mL plating was performed on Day 0 (inoculum) and Days 1, 3, 5, 7, or longer (biofilm), as indicated in the figure legend.

### Bacterial growth analysis

The growth assay was performed in 96-well plate in three media types MB, VNSS and 1% MB in ASW using LogPhase plate reader reading every 20 minutes at 600 nm (BioTek Instruments Inc.). Non-coated 24-well polystyrene plates were used as multi-well plates (ThermoFisher, USA). The data from three biological replicates with two technical replicates in each were used and smoothed using Savitzky-Golay to generate the growth curves in the Supplementary Fig. 3a. The maximum growth rate and lag phase were calculated using the Baranyi model^67^.

### Spotting assay and interaction analysis

The cell density of bacterial culture was adjusted as described in the top-down assembly. Plates were prepared by pouring 20 mL of the respective media into 10 cm diameter petri plates. Spots were created by spotting 5 μL of adjusted culture on the agar media plate, placing the plate on the printed layout, to equally space the spots. The plates were incubated at 30°C once the spots were absorbed. The cell phone image of the plates was used to quantify colony area and pixels using Image J Fiji^68^ which was then used to calculate the interaction strength using the workflow described in Liu et al^69^.

### Phylogenetic relatedness

The V3-V4 and full 16S sequences were analyzed for phylogenetic relatedness using the Maximum Likelihood method and the Tamura-Nei model in MEGA 11^70^ to generate a scaled, consensus tree from 1,000 bootstrap replicates.

### Chitinase assay

The three substrates (i) 4-Methylumbelliferyl N,N′-diacetyl-β-D-chitobioside (ii) 4-Methylumbelliferyl N-acetyl-β-D-glucosaminide and (iii) 4-Methylumbelliferyl β-D-N,N′,N′′-triacetylchitotriose suitable for chitobiosidase activity, β-N-acetylglucosaminidase activity and endochitinase activity, respectively, were used according to instructions in the fluorometric assay kit (Sigma-Aldrich; CS1030). Chitinase assay was performed using 10 μl of biofilm extract or effluent immediately frozen at −80°C after collection until the assay. A 30-minute incubation was performed at 37°C, and the end point fluorescence was measured with a Gen5 plate reader (BioTek Instruments Inc.). Unit definition: One unit of chitinase activity will release 1 mmole of 4-methylumbelliferone from the appropriate substrate per minute at pH 5.0 at 37°C.

### Water contact angle measurement

A 50 μL droplet of a bacterial culture in exponential phase was spotted on 1.5% hMA plates and incubated at 30°C for 24 h – 48 h until full growth (agar is hidden by compact growth where the droplet was placed). A square piece of agar, retaining 0.5 cm around the bacterial colony, was cut, and the water contact angle was measured using a Kruss drop shape analyzer (Kruss-scientific) with 2 μL of distilled water deposited on top of the bacterial biofilm at 20°C, and imaged at 2 seconds of contact.

### OCT imaging and image analysis

Using Thorlabs Ganymede OCT, a 5×5×0.2 mm 3D image of biofilm was captured from a coupon with biofilm submerged in 1 mL PBS to prevent biofilm drying during imaging. The OCT resolution is ~ 3 μm, leading to the exclusion of single-layer adhesion in the image. An image processing pipeline was developed based on development kits provided by ThorLabs, by adding (i) coupon surface estimation, which corrects for any curvature resulting from imaging in immersed condition, and (ii) robust de-noising algorithm which includes adaptive column-wise thresholding, adaptive cutoff heights, and median filtering, to make the pipeline more robust to background noise and artifacts, such as reflection.

### Data collection and statistical analysis

The initial experimental data collected were entered into Microsoft Excel. Statistical analysis and figures were generated using GraphPad Prism 10, R 4.4.3 and Python v3.12.7.

## Supplementary Material

Supplementary Files

This is a list of supplementary files associated with this preprint. Click to download.

- SupplementrayTable4.xlsx

## Figures and Tables

**Table 1:** Isolates used in the study

Taxonomic ID of isolates selected	Source	Isolate (Figure codes)	Trait analysis				Experimental		
			chit	adh	col-res	drag-red	chit	adh	other
*Alteromonas oceani* S35	^32^	H02					F	T	
*Marinobacter adhaerens* HP15	^32^	H13	T	T	F	F	F	T	
*Marinobacter dokdonensis* DSW-8	^32^	H22					F	T	
*Psychrobacter piscatorii* T-3–2	^32^	H25					F	T	cold-tolerant
*Psychrobacter piscatorii* T-3–2	^32^	H26					F	T	cold-tolerant
*Psychrobacter piscatorii* T-3–2	^32^	H27					F	T	cold-tolerant
*Psychrobacter piscatorii* T-3–2	^32^	H29					F	T	cold-tolerant
*Pseudoalteromonas* sp. ATL1	^33^	ATL1	T	T	F	T	T		
*Pseudoalteromonas piscicida* ATL3	^33^	ATL3	T	T	F	T	T		
*Rheinheimera* sp. ATLC3	^33^	ATLC3	T	T	F	F	T		
*Simiduia agarivorans* ATLC8b	^33^	ATLC8b	F	T	F	F	T		
*Pseudoalteromonas piscicida* ATL28	^33^	ATL28	T	T	F	T	T		
*Isoptericola dokdonensis* ATLC11	^33^	ATLC11					T		gram-positive
*Phaeobacter gallaeciensis* BS107	^38^ *	B13	F	T	F	F	F		antibiotic-producing; colonizer
*Phaeobacter* sp. Y41	^39^ *	B27	F	T	T		F		antibiotic-producing; colonizer
*Roseobacter denitrificans* Och 114	^40^ *	B2					F		primary colonizer
*Dinoroseobacter shibae* DFL12	^41^ *	B11					F		primary colonizer

chit = chitinase producing; adh = adhesion; col-res = colonization resistance; drag-red = drag reducing.

* Purchased from National Center for Marine Algae and Microbiota (https://ncma.bigelow.org). T = True, F = False. Empty cells indicate that the corresponding isolates are not selected in the trait analysis or are not analyzed in the experimental category.

## Data Availability

All data supporting the findings of this study are available within the paper and its Supplementary Information. The codebase for image analysis is available here: https://github.com/ZeyuT/EcoView.

## Code availability

The codebase for image analysis is available here: https://github.com/ZeyuT/EcoView. The remaining codes were previously reported, as described in the respective method section, and modified only for our data.
